# Genome-Wide Identification of Molecular Pathways and Biomarkers in Response to Arsenic Exposure in Zebrafish Liver

**DOI:** 10.1371/journal.pone.0068737

**Published:** 2013-07-29

**Authors:** Hongyan Xu, Siew Hong Lam, Yuan Shen, Zhiyuan Gong

**Affiliations:** Department of Biological Sciences, National University of Singapore, Singapore, Singapore; East Carolina University, United States of America

## Abstract

Inorganic arsenic is a worldwide metalloid pollutant in environment. Although extensive studies on arsenic-induced toxicity have been conducted using *in vivo* and *in vitro* models, the exact molecular mechanism of arsenate toxicity remains elusive. Here, the RNA-SAGE (serial analysis of gene expression) sequencing technology was used to analyse hepatic response to arsenic exposure at the transcriptome level. Based on more than 12 million SAGE tags mapped to zebrafish genes, 1,444 differentially expressed genes (750 up-regulated and 694 down-regulated) were identified from a relatively abundant transcripts (>10 TPM [transcripts per million]) based on minimal two-fold change. By gene ontology analyses, these differentially expressed genes were significantly enriched in several major biological processes including oxidation reduction, translation, iron ion transport, cell redox, homeostasis, etc. Accordingly, the main pathways disturbed include metabolic pathways, proteasome, oxidative phosphorylation, cancer, etc. Ingenity Pathway Analysis further revealed a network with four important upstream factors or hub genes, including Jun, Kras, APoE and Nr2f2. The network indicated apparent molecular events involved in oxidative stress, carcinogenesis, and metabolism. In order to identify potential biomarker genes for arsenic exposure, 27 out of 29 up-regulated transcripts were validated by RT-qPCR analysis in pooled RNA samples. Among these, 14 transcripts were further confirmed for up-regulation by a lower dosage of arsenic in majority of individual zebrafish. Finally, at least four of these genes, *frh3* (ferrintin H3), *mgst1* (microsomal glutathione S-transferase-like), *cmbl* (carboxymethylenebutenolidase homolog) and *slc40a1* (solute carrier family 40 [iron-regulated transporter], member 1) could be confirmed in individual medaka fish similarly treated by arsenic; thus, these four genes might be robust arsenic biomarkers across species. Thus, our work represents the first comprehensive investigation of molecular mechanism of asenic toxicity and genome-wide search for potential biomarkers for arsenic exposure.

## Introduction

Arsenic is a worldwide and mobile metalloid pollutant in environment. In nature, arsenic sulfides is converted to arsenic trioxide with rock weathering and enters into the arsenic cycle either as dust or by dissolution in rain, rivers or groundwater [1]–[3]. The majority of arsenic entering the organisms is in the pentavalent inorganic form As(V) via a simple diffusion mechanism [4] and is immediately reduced to trivalent arsenic As(III). Then organic and inorganic forms of arsenic are expelled in urine after several days, although some remain in the body for months or even longer [5], [6]. It is widely accepted that chronic exposure to inorganic arsenic can cause various diseases including cancers in skin, bladder, liver, kidney and lung [7], [8], diabetes [9], skin lesions (dyspigmentation, keratosis), peripheral vascular diseases, as well as disorders in the reproduction and nervous systems [10]–[12]. Numerous studies have been conducted to demonstrate the cellular and molecular mechanisms underlying the arsenic-induced pathology in human, indicating the association of environmental arsenic exposure with the development of diseases [1], [12]–[14].

However, although the data from some laboratory studies support the incidence and clinical symptoms of arsenic-induced diseases, available experimental data are insufficient to explain the epidemiological findings because of the limitation of technology and experimental models so far used [15]. To understand the clinical syndromes of arsenic-caused human diseases, it is important to use in vivo animal models. The zebrafish (*Danio rerio*) has become a popular vertebrate model system in toxicology and human disease studies because of its several inherent features: ease of obtaining a large number of animals for a statistical advantage, low animal husbandry cost for large-scale studies, and well established genetic and genomic tools including the availability of the complete genome sequence. Furthermore, genomic studies have proven that zebrafish and human generally share a common set of genes with well conserved gene synteny [16]–[19] and many important genes in development and molecular pathways including carcinogenesis are highly conserved and similarly regulated between zebrafish and human [20], [21].

Previously, our group has used a spotted DNA microarray platform, which included less than half of zebrafish genes in the genome, to analyse molecular response to arsenic exposure in zebrafish and provided preliminary evidence that arsenic can aberrantly regulate a series of genes associated with arsenic metabolism and oxidation, leading to DNA and protein damage, and thus cellular injury [22]. Now with the recently developed RNA sequencing technology, it is feasible to carry out genome-wide investigation of transcriptomic changes to toxicant insults as RNA sequencing is capable of measuring transcript abundance without the limitations of the predefined array content [23]–[25]. Furthermore, this analysis will not only help identify new biomarker genes for arsenic exposure but also allow us to analyse arsenic-altered biological pathways and potentially associated disease status. Currently there is no report for using the RNA sequencing platform to analyse transcriptomic response to arsenic exposure from either in vitro or in vivo models. In the present study, we employed the RNA sequencing platform to re-examine the molecular response of zebrafish liver to arsenic exposure and carry out detailed transcriptomic analyses for further understanding of molecular toxicity. We found that several important biological processes were perturbed by arsenic exposure, including oxidation reduction, translation, iron ion transport, cell redox and homeostasis, as well as related pathways in metabolism and diseases. Furthermore, as there are currently no biomarker genes available for predicting arsenic exposure, we took the advantage of RNA sequencing platform to identify most suitable biomarker genes from top responsive genes to arsenic exposure. We first validated these top responsive genes by RT-qPCR in zebrafish and then in Japanese medaka (*Oryzias latipes*) at individual fish level for more robustly responsive genes across different fish species.

## Materials and Methods

### Fish

Adult zebrafish (3-month old) were purchased from a local aquarium farm (Mainland Tropical Fish Farm, Singapore) and acclimated for at least one week in the departmental aquarium prior to chemical treatments. Medaka fish were bred in house and 3-month-old adult fish were used in the experiment. Experimental procedures were carried out following the approved protocol by Institutional Animal Care and Use Committee of National University of Singapore (Protocol 079/07).

### Chemical Exposure

As(V) or sodium arsenate (Na_2_HAsO_4_•7H2O) was purchased from Sigma-Aldrich (USA) and dissolved in water. As previously reported, LC50 of 96 h arsenic treatment in adult zebrafish was estimated to be within 20–25 ppm sodium arsenate [22]. In this study, we first used a relative high concentration (20 ppm) of sodium arsenate to treat zebrafish for RNA sequencing in order to ensure a toxicological response. Then we used a slight lower concentration (15 ppm) for more robust validation of arsenic responsive genes in zebrafish. For validation of biomarker genes in medaka, 20 ppm sodium arsenate was used as medaka were more tolerant to toxicant treatment based on our experience. For all treatment experiments, static exposure was conducted for only male fish at ambient temperature (28°C). The water and sodium arsenate were replaced daily and the fish were not fed throughout the 96-hour treatment.

### RNA Sample Preparation for SAGE Library Sequencing

Treated and untreated control fish were sacrificed and livers were collected from each fish. Total RNA was extracted using Trizol Reagent (Invitrogen). SAGE (series analysis of gene expression) RNA sequencing was carried out by Mission Biotech (Taiwan) using SOLiD™ Analyzer 4 (Applied Biosystems). Briefly, poly A RNA was purified using Dynabeads® Oligo(dT) EcoP (Invitrogen) and subjected to cDNA synthesis. Synthesized cDNA was digested by NlaIII and sequencing adapters were ligated to the cDNA fragments. All SAGE tags were mapped to the zebrafish Reference Sequence database (http://www.ncbi.nlm.nih.gov/RefSeq/) with allowance of maximum 2 mismatches and mapped tags for each transcript were normalized to TPM (tag counts per million). Only tags with unambiguous mapping to a single gene ID were considered in subsequent analyses.

### Differentially Expressed Genes and Gene Ontology Analysis

To analyse physiologically important genes, we selected transcripts with TPM>10 in at least one group of samples (control or treated) to be included for subsequent bioinformatic analyses. As it is impossible to get p-values from single samples, here we used fold-change (>2) to select differentially expressed genes, as previous studies showed that the actually measured magnitude of differential expression (fold change or ratio) is more consistent and reproducible in identifying differentially expressed genes than the statistical significance (p-value) [26], [27]. For calculation of fold changes, when a gene transcript was zero in one set of data, we assumed one SAGE tag was observed for the transcript and it was converted to p.2 TPM for a total of 0.2 TPM for a total of ∼5 million mapped tags in each sample. For the differentially expressed gene set, gene ontology (GO) analysis was conducted using DAVID (The Database for Annotation, Visualization and Integrated Discovery) [28] with the total zebrafish genome information as the background. Gene Ontology Fat categories were used for this analysis with a p-value cut-off of 0.05.

### Ingenuity Pathway Analysis (IPA)

To infer diseases and upstream factors associated with arsenic perturbation, IPA (Ingenuity Pathway Analysis) software (www.ingenuity.com) was used to analyse differentially expressed genes. Each gene identifier was mapped to its corresponding human homolog in the Ingenuity Pathways Knowledge Base. Out of the 1,444 differentially expressed genes, 1,025 genes were mapped to 921 human homologs and 707 genes were assigned to biological function analysis in the IPA database. For human homologs mapped with multiple zebrafish genes, the highest fold changes of zebrafish genes were used for subsequent functional implication analyses.

### Comparative Toxicogenomic Dataset (CTD) Analysis

The differentially expressed genes were further compared with CTD-curated genes/proteins that interact with arsenate (http://ctdbase.org/) [29], [30]. The CTD Batch Query tool was first used to retrieve all curated chemical-gene interactions for the term “sodium arsenate” and 1,129 genes were then retrieved in the dataset CTD_C009277_genes. MyGeneVenn was used to analyse the intersection between our differential expressed gene set and the CTD_C009277_genes. Enriched pathways were identified by Gene Set Enricher available from CTD website http://ctdbase.org/tools/enricher.go).

### RT-qPCR

Total RNA was extracted from livers of arsenic-treated and untreated zebrafish or medaka. 2 µg of DNase-treated total RNA was used for synthesizing first strand cDNA using an oligo-dT primer and SuperScript^TM^II Reverse Transcriptase according to the manufacturer's protocol (Invitrogen). The cDNA samples were used for qPCR analysis using Lightcycler-FastStart DNA Master SYBR Green 1 and Lightcycler480 (Roche Applied Science) according to the manufacturer’s instruction. In our unpublished experiments, seven housekeeping genes (*rnf7, rplp2*, *rppl13a, rplp0, b-actin1, hrp1 and b2m*) were compared as internal control under different chemical treatment conditions and the geometric mean of three genes (*rnf7, rplp2* and *rppl13a*) was used as the normalized factor since it was evaluated as the best internal control by the the software GeNorm Plus [31]–[33]. The −ΔΔCt represents the expression level of genes, the statistical comparison of the relative mean expression level for each gene between test and control groups was performed using Student’s T-test with P-value<0.05 being considered significant. The cluster figure was generated by software Mev4.

## Results

### Gene Ontology Analysis of Differentially Expressed Genes Caused by Arsenic Exposure

**Figure 1:**
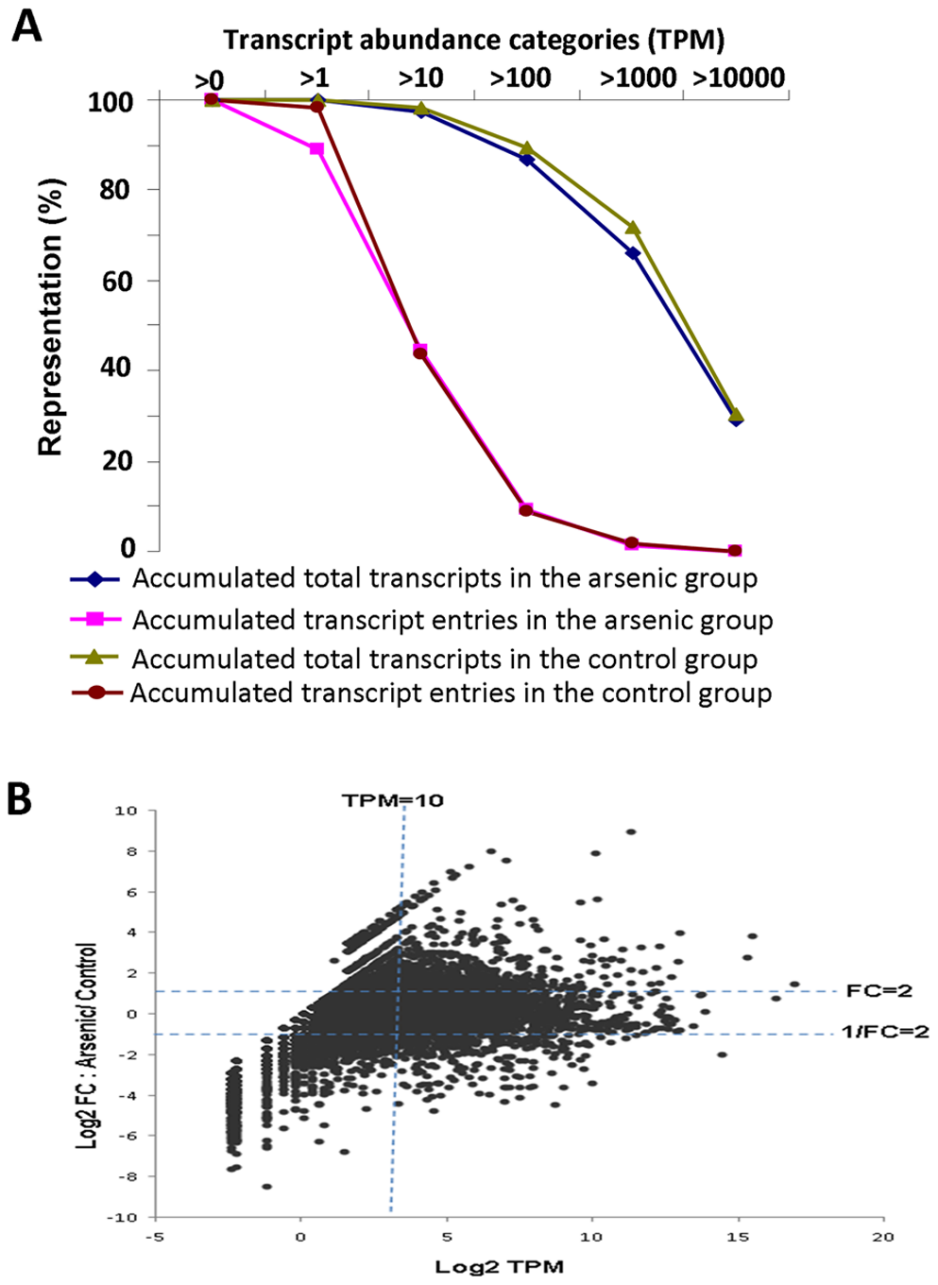
Comparison of transcriptomic profiles between arsenic-treated and control groups. (A) Distribution of transcript entries and total transcript counts in both arsenic-treated and control groups. The percentages of accumulated transcript counts or transcript entries are plotted over different transcript abundance categories. (B) Plot of transcript change fold (Y-axis) versus transcript TPM (X-axis) after arsenic exposure. Both axes are in log2 scale and TPM in the X-axis is based on the treatment group.

Totally, 27.2 million SAGE tags, 13.6 million each from arsenic-treated and untreated control samples, were obtained from RNA sequencing. After mapping to the zebrafish Reference Sequence database, 5.6 million and 6.9 million uniquely mapped tags were mapped to 8,318 and 8,434 gene entries, respectively, from the arsenic-treated and untreated groups. The RNA-seq data were submitted to Gene Expression Omnibus with an access number GSE48427). The distribution of tag entities and total tag counts over different tag abundance categories are shown in Figure 1A and the arsenic-treated and untreated groups had very similar profiles. The abundance of transcripts from the two groups ranged from 0.2 to 130,132 TPM in abundance. In general, a low percentage of gene entries have a high TPM but these high abundant transcripts constitute the majority of mRNA transcriptome. For example, only 759 (9%) and 760 (9%) gene entries have TPM above 100, but these transcripts constitute 87% and 89% of transcriptome respectively in the arsenic-treated and untreated control groups.

To elucidate the molecular response of zebrafish livers to arsenic exposure, RNA-SAGE data were subjected to a series of bioinformatic analyses as outlined in Figure S1. First, 1,444 differentially expressed genes, including 750 up-regulated genes and 694 down-regulated genes by arsenic treatment (Table S1), were identified using criteria of fold change ≥2 and TPM≥10 in either sample (Fig. 1B). The selection of TPM>10 was for analyses of physiologically significant transcripts as 10 TPM is equivalent to 3.3 transcripts per cell [34], [35]. A total of 3,703 and 3,661 genes had TPM above 10 respectively in the arsenic-treated and untreated samples and these transcripts constitute about 97% of mRNA transcriptome (Fig. 1A).

GO analysis of the 1,444 differentially expressed genes, in which 1,211 genes were mapped to identifiers in DAVID Knowledgebase, showed significant enrichment in several categories (Table 1). In the Biological Process, the top enrichment terms were oxidation reduction, translation, homeostatic process, cellular homeostasis, proteolysis, iron ion transport, cell redox and homeostasis. Accordingly, in Molecular Function, iron ion binding appeared to be the top of the list while structural constituent of ribosome and many other categories apparently related to oxidation reduction and metabolism were also enriched. In Cellular Component, mitochondrion appeared at the top of the list, further highlighting the importance of oxidation reduction in response to arsenic exposure (Table 1).

**Table 1:** Significantly affected gene ontology terms by arsenic exposure.

Category	Term	Count	Enrichment	p-Value
**Biological**	GO:0055114∼oxidation reduction	78	1.79	3.330E-04
**Process**	GO:0006412∼translation	48	2.14	3.759E-04
	GO:0042592∼homeostatic process	33	2.42	1.384E-03
	GO:0019725∼cellular homeostasis	25	2.56	7.568E-03
	GO:0006508∼proteolysis	73	1.54	3.378E-02
	GO:0006826∼iron ion transport	8	5.79	3.821E-02
	GO:0045454∼cell redox homeostasis	15	3.10	3.473E-02
**Molecular**	GO:0005506∼iron ion binding	48	2.25	8.657E-05
**function**	GO:0003735∼structural constituent of ribosome	30	2.49	1.759E-03
	GO:0005198∼structural molecule activity	51	1.89	2.429E-03
	GO:0070011∼peptidase activity, acting on L-amino acid peptides	62	1.73	2.944E-03
	GO:0008233∼peptidase activity	63	1.68	5.511E-03
	GO:0008235∼metalloexopeptidase activity	10	4.72	1.218E-02
	GO:0004866∼endopeptidase inhibitor activity	18	2.83	1.112E-02
	GO:0008199∼ferric iron binding	7	6.89	1.555E-02
	GO:0008238∼exopeptidase activity	13	3.34	2.094E-02
	GO:0030170∼pyridoxal phosphate binding	13	3.20	2.872E-02
	GO:0070279∼vitamin B6 binding	13	3.20	2.872E-02
	GO:0004298∼threonine-type endopeptidase activity	9	4.43	2.880E-02
	GO:0070003∼threonine-type peptidase activity	9	4.43	2.880E-02
	GO:0030414∼peptidase inhibitor activity	18	2.53	2.695E-02
	GO:0016769∼transferase activity, transferring nitrogenous groups	10	3.81	3.457E-02
	GO:0004857∼enzyme inhibitor activity	20	2.27	4.203E-02
**Cellular**	GO:0005739∼mitochondrion	50	2.25	1.033E-05
**component**	GO:0005840∼ribosome	35	2.49	7.856E-05
	GO:0005829∼cytosol	26	2.58	1.094E-03
	GO:0030529∼ribonucleoprotein complex	41	1.86	6.125E-03
	GO:0000502∼proteasome complex	14	3.46	5.464E-03
	GO:0044429∼mitochondrial part	29	2.10	7.570E-03
	GO:0005839∼proteasome core complex	9	4.54	1.565E-02
	GO:0043232∼intracellular non-membrane-bounded organelle	79	1.42	1.907E-02
	GO:0043228∼non-membrane-bounded organelle	79	1.42	1.907E-02
	GO:0005740∼mitochondrial envelope	25	2.07	1.949E-02

Note: Count is number of genes involved. Fold Enrichment is (m/n)/(M/N), where **N** = all genes in zebrafish, **M** = all genes belonging to a GO term, **n** = genes from the differentially expressed gene set, **m** = genes from the differentially expressed gene set belonging to a GO term.

### Predicted Diseases and Transcription Factors Perturbed by Arsenic

**Figure 2:**
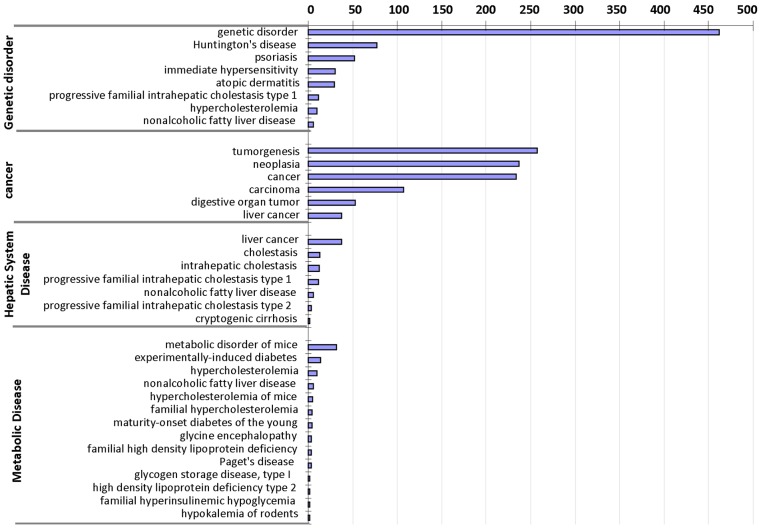
Diseases inferred by IPA based on differentially expressed genes after arsenic exposure. The bar chart shows the number of arsenic-deregulated genes matched in different disease or disorder categories and only top significant categories (P<0.01) were selected to show. The same gene may be assigned to more than one categories.

To further elucidate arsenic-induced transcriptomic changes, IPA software were used to analyse the differentially expressed genes to identify associations with disease conditions. Out of the 1,444 differentially expressed genes, 1,025 genes could be mapped to 921 human homologs and 707 genes were assigned to biological function analysis in the IPA database. The major and significant diseases or disorders included Genetic Disorder (462 genes), Cancer (302 genes), Metabolic Disease (65 genes), Hepatic System Disease (55 genes), Endocrine System Disorders (28 genes). A more detailed examination of the sub-functional annotation showed that Genetic Disorder included immediate hypersensitivity, hypercholesterolemia and nonalcoholic fatty liver disease. Many genes were enriched in several cancer categories, including tumorgenesis, neoplasia, carcinoma, digestive organ tumor and liver cancer. Metabolic Disease included experimentally-induced diabetes, hypercholesterolemia, nonalcoholic fatty liver disease, etc, while Hepatic System Disease included liver cancer and cholestasis (Figure 2). Thus, arsenic exposure has a broad effect on liver function, leading to several metabolic disorders and potentially cancer, consistent with the findings from previous reports that liver is a major target of arsenic toxicity and liver cancer could be induced by chronic exposure to arsenic [7], [8], [36].

**Figure 3:**
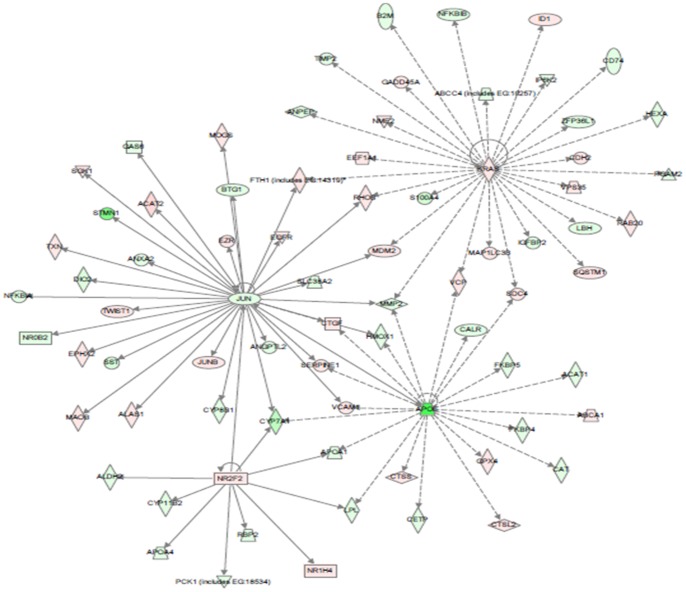
Key upstream regulator networks modulated by arsenic exposure. The upstream network was generated by IPA and the networks indicate predicted upstream regulators and their downstream target genes presented in the differentially expressed gene set. Up-regulated genes are in red and down-regulated in green. Solid arrow lines represent direct interaction while dotted lines indirect intereaction.

To further analyse molecular relevance to altered biological process and relevant diseases, IPA upstream factor analysis was carried out and a network with four hub factors (Nr2f2, Jun, Kras and ApoE) is presented in Figure 3. The network is apparently related to oxidative stress (Nr2f2), cancer (Jun, Kras) and metabolic pathways (ApoE). As displayed in the network, these upstream factors regulate their downstream factors respectively or cooperatively. For example, Jun regulated the expression of *fth1, rhob, mdm2, mmp2, ctcf, serpene1, vcam1, cyp7a1, acat1* and other genes. Of these Jun-regulated genes, *fth1, rhob, mdm2* and *mmp2* might also interact with Kras while *mmp2, ctcf, serpne1, vcam1* and *cyp7a1* interacted with ApoE. The relationship of these factors and their interaction are further elaborated in Discussion.

### Comparative Toxicogenomic Analysis

To define the robust and conserved pathways and genes regulated by arsenic across species, a comparative toxicogenomic analysis was conducted between our current study and CTD database, in which 1,129 genes were curated as chemical-gene interactions under the term “sodium arsenate” (CTD_C009277_genes). Intersection analysis of this set of genes and differentially expressed gene set from the current study showed 84 common arsenic-responsive genes (Table S2). However, among the top 239 genes based on fold changes (FC≥8) in our current study (Table S1), only 8 genes (three up-regulated: *gsr*, *klf11, gsto1*; five down-regulated: *hsd11b2, gsto1, hsd11b2, aldh2, aqp3, dio2* and *krt8*) were reported in the CTD dataset (Table S2), indicating that many of the top arsenic-responsive genes have not been captured by CTD and hence there is an importance of the re-examination of arsenic-caused transcriptomic changes using the RNA sequencing technology.

To elaborate how the arsenic-responsive genes function through molecular interactions in related diseases, the 1,444 differentially expressed genes were analysed Gene Set Enricher in the CTD database. A total of 36 significantly enriched KEGG (Kyoto Encyclopaedia of Genes and Genomes) pathways were identified based on 920 genes recognized by Gene Set Enricher. However, only less than half (17) of these enriched pathways have been reported in the arsenic CTD dataset (CTD_C009277_pathways) and 19 pathways were newly identified in the present study, including the top two most significantly altered pathways, Metabolic pathways, Proteosome (folding, sorting & degradation) and many others such as Protein processing in endoplasmic reticulum, PPAR signaling pathway, Pathways in cancer, MAPK signaling pathway, Hepatitis C, etc. (Table 2).

**Table 2:** Enriched KEGG pathways identified by Gene Set Enricher of CTD.

Pathways	Pathway ID	P-value	Gene counts
*Metabolic pathways	KEGG:01100	3.99E-49	142
*Proteasome (folding, sorting & degradation)	KEGG:03050	1.58E-11	16
Glycine, serine and threonine metabolism	KEGG:00260	1.05E-08	12
Glycolysis/gluconeogenesis	KEGG:00010	1.10E-06	14
*Arginine & proline metabolism	KEGG:00330	1.41E-06	13
*Glutathione Metabolism	KEGG:00480	4.05E-06	12
*Protein processing in endoplasmic reticulum	KEGG:04141	4.20E-06	21
Tryptophan metabolism	KEGG:00380	7.15E-06	11
*PPAR signaling pathways	KEGG:03320	2.02E-05	13
Oxidative phosphorylation	KEGG:00190	2.79E-05	17
Ribosome	KEGG:03010	7.19E-05	14
*Pathways in cancer	KEGG:05200	9.58E-05	27
*MAPK signaling pathway	KEGG:04010	1.77E-04	24
*Hepatitis C	KEGG:05160	2.52E-04	16
Purine metabolism	KEGG:00230	3.45E-04	18
Bladder cancer	KEGG:05219	3.68E-04	9
Proximal tubule bicarbonate reclamation	KEGG:04964	4.41E-04	7
Peroxisome	KEGG:04146	4.95E-04	12
Propanoate metabolism	KEGG:00640	6.76E-04	8
Fat digestion and absorption	KEGG:04975	0.00143	9
*Ubiquitin mediated proteolysis	KEGG:04120	0.00162	15
Antigen processing and presentation	KEGG:04612	0.00192	11
*Cardiac muscle contraction	KEGG:04260	0.00218	11
*Cell cycle	KEGG:04110	0.00261	14
*Mineral absorption	KEGG:04978	0.00281	9
*Endometrial cancer	KEGG:05213	0.0033	9
Parkinson's disease	KEGG:05012	0.00341	14
*Huntington	KEGG:05016	0.00381	17
Complement and coagulation cascades	KEGG:04610	0.00447	10
*Leukocyte transendothelial migration	KEGG:04670	0.0046	13
*Carbohydrate digestion and absorption	KEGG:04973	0.00522	8
Pyrimidine metabolism	KEGG:00240	0.00549	12
TCA cycle	KEGG:00020	0.00609	7
Pyruvate metabolism	KEGG:00620	0.0062	8
*Prostate cancer	KEGG:05215	0.00856	11
*Insulin signaling	KEGG:04910	0.00914	14

Note: Pathways indicated with asterisks are newly identified in the present study.

### Potential Biomarker Genes for Arsenic Exposure

**Figure 4:**
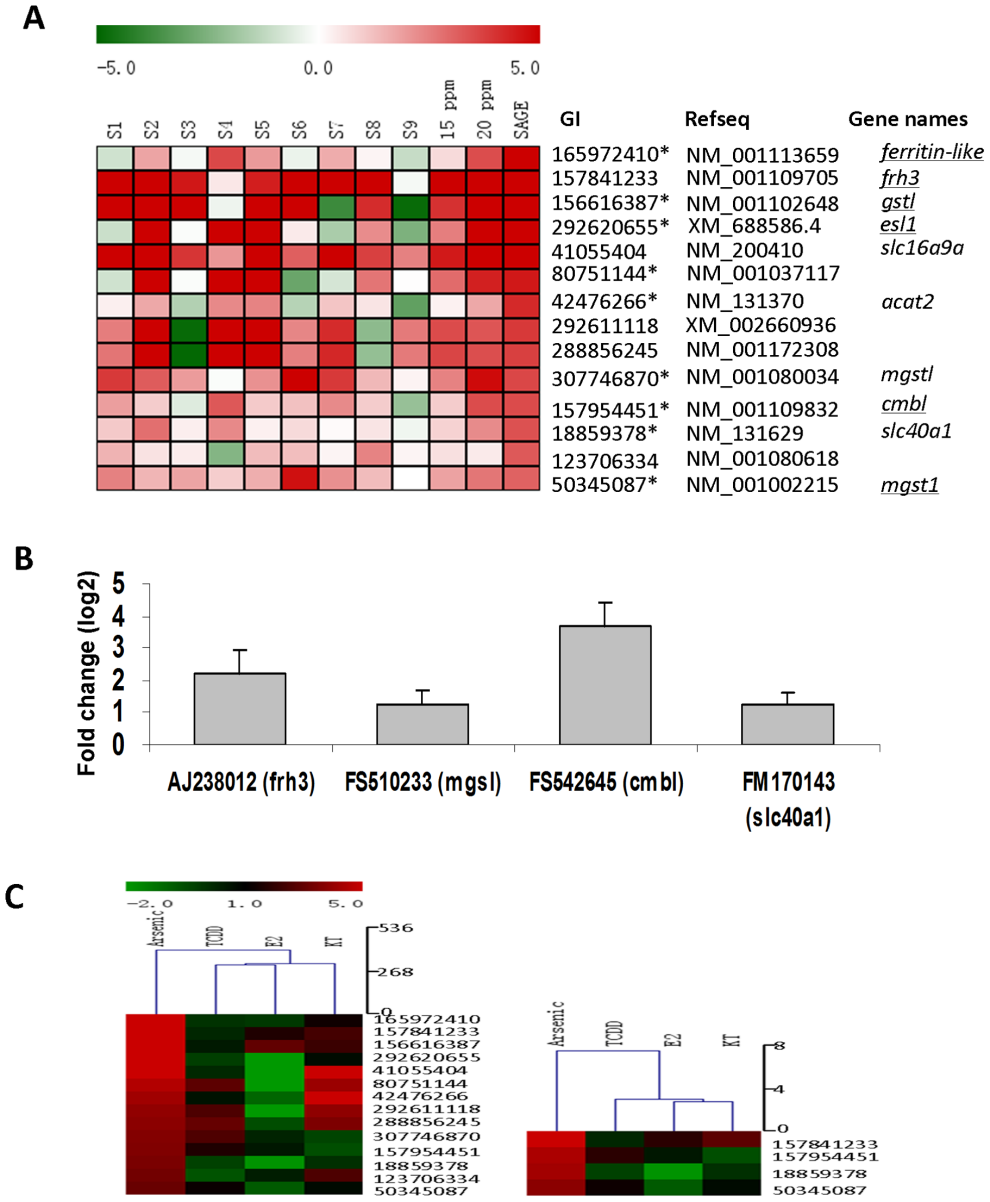
Preliminary identification of potential biomarker genes for arsenic exposure. Selected up-regulated genes by arsenic exposure were examined by RT-qPCR in individual zebrafish (A) and medaka (B) after treatment with arsenic. (A), Fold changes (log2 ratio) of 14 up-regulated genes measured by RT-qPCR. S1-S9, 9 individual zebrafish treated with 15 ppm sodium; 15 ppm, average of the 9 individual fish; 20 ppm, RT-qPCR measurement from the pooled RNA sample used for RNA-SAGE sequencing; SAGE, RNA-SAGE data for comparison (Log2 fold change). The 9 genes displayed dosage-dependent effect between 15 ppm and 20 ppm are indicated with asterisks. Zebrafish gene symbols and names are shown based on NCBI and underlined genes are annotated manually. (B), Average of fold changes (log2 ratio) of four validated medaka genes in 4 individual medaka fish. (C) Comparison of the expression of arsenic biomarker genes in other chemical treatments by hierarchical clustering heatmap. RNA-SAGE data from the current study (Arsenic) were compared with hepatic RNA-SAGE data from zebrafish treated with 5 µg/L 17β-estradiol (E2), 5 µg/L 11-keto testosterone (KT11) or 10 nM 2,3,7,8-tetrachlorodibenzo-*p*-dioxin (TCDD). The left clustering is based on the 14 genes identified from zebrafish and the right based on the four genes from both zebrafish and medaka.

Another important motivation of the current study is to identify reliable and robust biomarkers suitable for arsenic exposure by genome-wide search. Here we first tested top up-regulated genes (55 genes having fold change >8 and TPM>30) for RT-qPCR validation. Among the top 55 up-regulated genes, 29 of them were successfully amplified by PCR while the rest 26 genes could not be analysed either because of insufficient sequences for specific PCR primers or due to the failure of generating PCR products. Finally 27 genes were confirmed to be up-regulated using the same pooled RNA sample for RNA-SAGE sequencing, indicating the consistency of the two technical platforms (Table S3). To analyse the applicability of these genes as biomarkers, we further validated their changes at individual fish level from another set of fish liver samples treated with a lower concentration of sodium arsenate (15 ppm) in order to identify more robustly responsive genes. Among the nine individual fish tested, 14 out of the 27 genes could be further validated in at least five individual fish (Figure 4A). 9 out of the 14 genes showed dosage-dependent response by comparison their RNA levels between 15 and 20 ppm. Intriguingly, among these 14 genes, only 4 of them (*slc16a9a, acat2, mgstl* and *slc40a1*) were annotated in the Zebrafish Reference Sequence database. By manual sequence analyses, we annotated another six transcripts based on the available cDNA sequences in Genbank (*ferritin-like* [NM_001113659], *frh3* [NM_001109705], *gstl* [NM_00102648], *es1l* [XM_688586.4], *cmbl* [NM_001109832] and *mgst1* [NM_001002215.1]). However, there are still four unannotated or potentially novel genes in zebrafish suggesting that there are still novel arsenic-responsive genes that are being uncovered from the RNA-seq study.

To further test the putative biomarker genes and their applicability in other fish species, we performed homolog/ortholog search of these genes in medaka, which is about 3 million years divergent from zebrafish in evolution [37]. By gene sequence alignment and shared synteny analysis, we found medaka orthologues/homologues of 9 genes from the 13 validated zebrafish genes. By RT-qPCR analysis of four individual fish samples similarly treated with arsenic, four medaka genes were significantly up-regulated by arsenic in at least three out of four medaka fish (Fig. 4B). These four genes were *frh3* (ferrintin H3), *mgst1* (microsomal glutathione S-transferase-like), *cmbl* (carboxymethylenebutenolidase homolog) and *slc40a1* (solute carrier family 40 [iron-regulated transporter], member 1). Interestingly, *slc40a1* was also observed to be up-regulated by arsenic exposure in our previous microarray analysis [22] while the other three genes are reported to respond arsenic exposure in animals for the first time.

To address the question whether the 14 biomarker genes identified from zebrafish provide specificity for predicting arsenic toxicity, we compared the available RNA-SAGE data from other chemical treatments including 17β-estradiol (E2) [38], 11-keto testosterone (KT11) [38] and 2,3,7,8-tetrachlorodibenzo-*p*-dioxin (TCDD) (our unpublished data). We found that only the arsenic treated sample showed high levels of up-regulation of all of the 14 biomarker genes. Hierarchical clustering indicated that use of the 14 biomarker genes, arsenic-treated samples could be easily separated from the rest of samples (Figure 4C). Furthermore, using the four biomarker genes common to both zebrafish and medaka, the distinction of arsenic-treated samples was even more prominent as the four genes were most down-regulated by other treatments (Figure 4D). These comparisons indicate the potential specificity of these biomarker genes for identification of arsenic toxicity.

## Discussion

### Biological Processes Perturbed by Arsenic Exposure

The present study represents the first comprehensive analysis of genome-wide response to arsenic exposure in an *in vivo* biological model. Arsenic exposure caused a change of the expression of a large number of genes and these differentially expressed genes were inferred to be mainly involved oxidation reduction, translation, homeostatic process, iron ion binding and transport by GO analysis (see Table 1 for complete list).

The oxidation reduction process was up-regulated, suggesting potential oxidative stress induced by arsenic exposure in the zebrafish liver, which is consistent with previously reports that oxidative stress is an important initiating factor in the pathogenesis of arsenic-induced liver injury [39], [40]. Furthermore, oxidative stress would subsequently alter the expression of genes involved in cellular homeostasis [41] and increased production of oxidizing species leading to glutathione depletion [42]. Consistent with our data, it has been reported from arsenic-fed mice that there is a decreased level of hepatic glutathione as well as decreased enzyme activities of glucose-6-phosphate dehydrogenase (G6pd) and glutathione peroxidase (Gpx) [43]. In contrast, cells resisting to arsenic toxicity in mammals correlate with higher levels of glutathione and glutathione-related enzymes including glutathione reductase (Gsr), glutathione S-transferase (Gst) and glutamate cysteine ligase (Gcl) [44]. Similarly, our RNA-SAGE data indicated that the mRNA level for *gpx8* (glutathione peroxidase 8) were decreased while genes encoding glutathione related enzymes including *gsr*, *gstm3* (glutathione S-transferase mu 3), *gsto1* (glutathione S-transferase omega 1), *gstt1b* (glutathione S-transferase theta 1 glutathione S-transferase theta 1b), *herpud1* (homocysteine-inducible, endoplasmic reticulum stress-inducible, ubiquitin-like domain member 1), *mgst1* (microsomal glutathione S-transferase 1) and *mgst3* (microsomal glutathione S-transferase 3) were up-regulated (Table S1, Table 2). In addition, several important cellular antioxidants were also deregulated, including up-regulated *prdx2* (peroxiredoxin 2), *mt2* (Metallothionein-2) and *gls2* (glutaminase 2), and down-regulated *cat* (catalase) (Table S1).

It is also well documented that iron or iron containing molecules interact with the oxidizing agents such as H_2_O_2_ and therefore play an important role in reducing cellular oxidative stress [45]. It has also been shown that intra-lysosomal iron chelation is able to reduce H_2_O_2_-induced DNA damage and apoptosis in cultured cells [46]–[48]. Consistent with this, one of the most significant molecular function identified from our transcriptomic analysis is iron ion binding. Iron is an essential constituent of living cells and organisms as it is involved in fundamental functions, including oxygen transport, DNA biosynthesis, transfer of electrons in the respiratory chain and xenobiotic metabolism [49]. Iron deficiency leads to anemia due to the depletion of iron store and thus insufficient haemoglobin in the blood. However, iron is also potentially toxic and excess iron promotes oxidative stress and tissue damage, resulting in complications such as liver fibrosis, hepatocellular carcinoma or diabetes [50], [51]. Therefore, it is understandable from our transcriptomic data analysis that arsenic exposure was associated with many genetic disorders, metabolic diseases, hepatic system diseases and even cancers through disruption of metabolic pathways, immune system, PPARa signaling pathway, and glutathione metabolism.

The hallmarks of iron deficiency and excess are determined by several critical proteins involved in iron binding, transport and recycle [52], [53]. In our study, a number of genes involved in iron binding and transport, such as *slc40a1* (solute carrier family 40 [iron-regulated transporter] member 1, also known as ferroportin 1), *fth1* (ferritin, heavy polypeptide 1) and *frh3* (ferrintin H3), were up-regulated by arsenic exposure, implying that the iron homeostasis of liver was perturbed. Moreover, both *slc40a1* and *fth1* have been previously reported to be associated with arsenate toxicity [22], [54]. *Slc40a1* encodes a cellular iron exporter to transport iron from inside a cell and is mainly expressed in macrophages, enterocytes, and hepatocytes [55]–[57]. *Fth1* encodes a heavy subunit of ferritin, a globular protein complex consisting of 24 subunits, which is the primary intracellular iron-storage protein in both prokaryotes and eukaryotes for keeping iron in a soluble and non-toxic form and thus acts as a buffer against iron deficiency and overloading. *Frh3* encodes an uncharacterized protein of 175 amino acid (NP_001103175) that contains a ferritin-like domain (NCBI domain ID: pfam00210) as it displays high identity (96%) with catfish ferritin H [58] and mammalian Ferritin [59]. The concentration of Ferritin increases in response to stresses including anoxia, pathogenesis and carcinogenesis [60]. For instance, it has also been reported that Ferritin binds and activates p53 under oxidative stress [61] and the overexpression of H-ferritin (Ferritin heavy subunit) promotes radiation-induced leukemia/lymphoma in mice [62]. Interestingly, both *frh3* and *slc40a1* were identified to be up-regulated by arsenic in both zebrafish and medaka in the present study (Figure 4).

Iron is mainly stored in macrophages of the reticulo-endothelial system and in hepatocytes [63], [64]. It has been proposed that a nuclear-encoded mitochondrial ferritin isoform is very likely involved in the storage of excess iron within the mitochondria [65] which, as a site for iron utilization and ROS (reactive oxygen species) production, are particularly vulnerable to oxidative stress. Iron may be mobilized from the ferritin complex to cytoplasm following localized protein unfolding [66] or degradation in lysosomes [67]. Ferritin may also undergo degradation by the proteasome, following iron depletion or oxidation [68]. Therefore, the differentially expressed genes of this study were significantly enriched as components of mitochondrion, ribosome and proteasome complex.

### Networks and Diseases Relevant to Arsenic Exposure

As reported from experimental and clinic investigations, inorganic arsenic can cause a variety of cancer [7], [8], diabetes [9] and other diseases. However, it remains largely unclear about the molecular events behind the pathogenesis of arsenic exposure. Our transcriptomic analysis may provide molecular insights associating the differentially expressed genes to cancers and metabolic diseases (Figure 2). Further analysis of upstream regulators and network revealed four hub factors, Nr2f2, Jun, Kras and ApoE (Figure 3). Moreover, many transcription factors, such as Prox1, Nr1d1, Rxrg, Hlf, Nr0b2, Foxm1, Nfn1a, Jun, Junb, Fox3, Etv5, Sqtm1, and NF-kB1b were differentially regulated by arsenic exposure in our data, consistent with reports in mammalian cells, where the AP-1 complex, NF-kB, and the MTF-1 (metal-responsive transcription factor 1) could be activated by arsenic [69]–[72].

As illustrated in Figure 3, Jun may regulate the expression of more than 30 genes. Among these, *fth1*, *rhob, mdm2* and *mmp2* are also regulated by Kras, another prominent oncoprotein [73]–[75]. Here, Kras may directly or indirectly regulate the activity of *abcc4, anpep*, *b2m*, *cd74*, *cdh2*, *eef1A1*, *fth1*, *gadd45A*, *hexa*, *id1*, *igfbp2*, *ip6k2*, *mdm2*, mmp2, *nfkbib*, *rab20*, *rhob*, and *sqstm1*. Most of these genes have been well documented for tumorgenesis and immune response. For example, Nfkbib (NF-kappa-B inhibitor beta) functions to inhibit the NF-κB transcription factor [76]. Deregulation of NF-κB has been linked to cancer, inflammatory and autoimmune diseases [77]–[79]. RhoA (Ras homolog gene family, member) is a small GTPase protein known to be involved in the regulation of cell division [80]. Rab20, another member of RAS oncogene family, is also up-regulated by Kras. Mdm2 (murine double minute, oncogene) is an important negative regulator of the p53 tumor suppressor [81], [82] and Mdm2 overexpression, in cooperation with oncogenic Ras, promotes transformation of primary rodent fibroblasts and tumorigenesis in nude mice [83]. In addition, Egfr (epidermal growth factor receptor) [84], Gadd45A (growth arrest and DNA-damage-inducible protein) [85] and Id1 (inhibitor of differentiation) [86], [87] are well known to function in cancer pathways. Interestingly we observed in this study that Jun and Kras appeared to directly up-regulate *fth1*, which acts as a buffer against iron deficiency and overloading [88]. This further explains the relationship between iron homeostasis and tumorgenesis, and illustrates the molecular mechanisms behind the previous reports that the concentration of Ferritin increases in response to stresses including anoxia [60], pathogenesis and carcinogenesis [89].

ApoE is essential for catabolism of triglyceride-rich lipoprotein constituents [90]. Here, the significant down-regulation of ApoE possibly reduced the expression of *apoa1, cyp7a1, calr, acat1, cat, cetp, fkbp4, fkbp5, mmp2* and *hmox1*, and increased expression of *ctss, ctsl2, gpx4, sdc4* and *apca1*. Some of these genes have been well documented and related to metabolism, especial to lipid metabolism. For example, Apoa is secreted into blood circulation on the surface of newly synthesized chylomicron particles and modulates the efficiency of enterocyte and hepatic transcellular lipid transport *in vitro* [91]. The altered expression of *cat* (catalase) is associated with oxidative DNA damage and subsequent cancer susceptibility [92], [93]. Cetp (Cholesteryl ester transfer protein), also called plasma lipid transfer protein [94], [95] and Abca1 (ATP-binding cassette transporter sub-family) are major regulators of cellular cholesterol and phospholipid homeostasis. Abca1functions as a cholesterol efflux pump in the cellular lipid removal pathway [96]. It also mediates the transport of lipids between Golgi and cell membrane. Since Abca1 is needed throughout the body, it is present highly in tissues involved in the turnover of lipids such as the liver and adipose tissue [96]. Overexpression of ABCA1 has been reported to induce resistance to curcumin, a dietary polyphenolic antioxidant with an anti-inflammatory role [97]. GPX4 (Glutathione peroxidase 4) in humans protects cells against membrane lipid peroxidation [98]. Therefore, through the effect of ApoE and its downstream genes, arsenic modulated the lipid metabolism in zebrafish liver. It is also interesting to note that the expression of *jun* might also be affected by ApoE, further highlighting the increasingly recognized relationship of carcinogenesis and metabolic disorders [90].

Nr2f2 (nuclear receptor subfamily 2, group F, member 2) is a member of the steroid thyroid hormone superfamily of nuclear receptors [99] and it appeared to be involved in the regulation of many different genes including *aldh2, nr1h4, apoa1, apoa4, cyp7a1, lpl, pck1, cyp11b2* and *rbp2* (Figure 3). Most of these genes are related to oxidative stress and drug metabolism. For example, *aldh2* encodes a member of the aldehyde dehydrogenase family and it is the second enzyme of the major oxidative pathway of alcohol metabolism [100]. Nr1h4 (nuclear receptor subfamily 1, group H, member 4) is a nuclear receptor and regulates the expression of genes involved in bile acid synthesis and transport [101]. Pck1, Phosphoenolpyruvate carboxykinase 1 (soluble), is a main control point for the regulation of gluconeogenesis [102]. Cyp11b2 (cytochrome P450, family 11, subfamily B, polypeptide 2) and Cyp7a1 (cytochrome P450, family 7, subfamily A, polypeptide 1) belong to the cytochrome P450 protein family and these monooxygenases catalyze many reactions involved in drug metabolism and synthesis of cholesterol, steroids and other lipids, that are related to cellular oxidative stress [103], [104].

In summary, our study indicates that arsenic could evoke a series of molecular events involved in oxidative stress, iron homeostasis, lipid metabolism disorder and carcinogenesis. The top significant molecular functions of differentially expressed genes included iron ion binding, structural constituent of ribosome and several types of peptidase activity (Table 1) which are related to oxidative stress, iron homeostasis and metabolism processes. However, whether these effects are mainly associated with arsenic or some of them may also be commonly shared by other metal toxicants will be an interesting question for future investigation using the RNA-seq based transcriptomic approach.

## Supporting Information

Figure S1 **Flowchart of the study.** Main steps and analyses are represented in each box in chronical order. Data generated from these analyses are shown in figures and tables as indicated in each box.
(PDF)

Table S1 **1,444 differentially expressed genes by arsenic treatment (Fold change >2, TPM>10).**
(XLSX)

Table S2 **84 arsenic-responsive genes common to this study and to CTD.**
(XLS)

Table S3 **Up-regulated genes validated by qPCR using the same RNA sample for RNA sequencing.**
(DOCX)
