# Supplementary material for: Genome-Wide Identification of Molecular Pathways and Biomarkers in Response to Arsenic Exposure in Zebrafish Liver
Source: PLoS One. 2013 Jul 29;8(7):e68737. doi: 10.1371/journal.pone.0068737 (PMC3726666; doi:10.1371/journal.pone.0068737)
Supplement: Figure S1 — Flowchart of the study. Main steps and analyses are represented in each box in chronical order. Data generated from these analyses are shown in figures and tables as indicated in each box. (PDF) [file pone.0068737.s001.pdf]

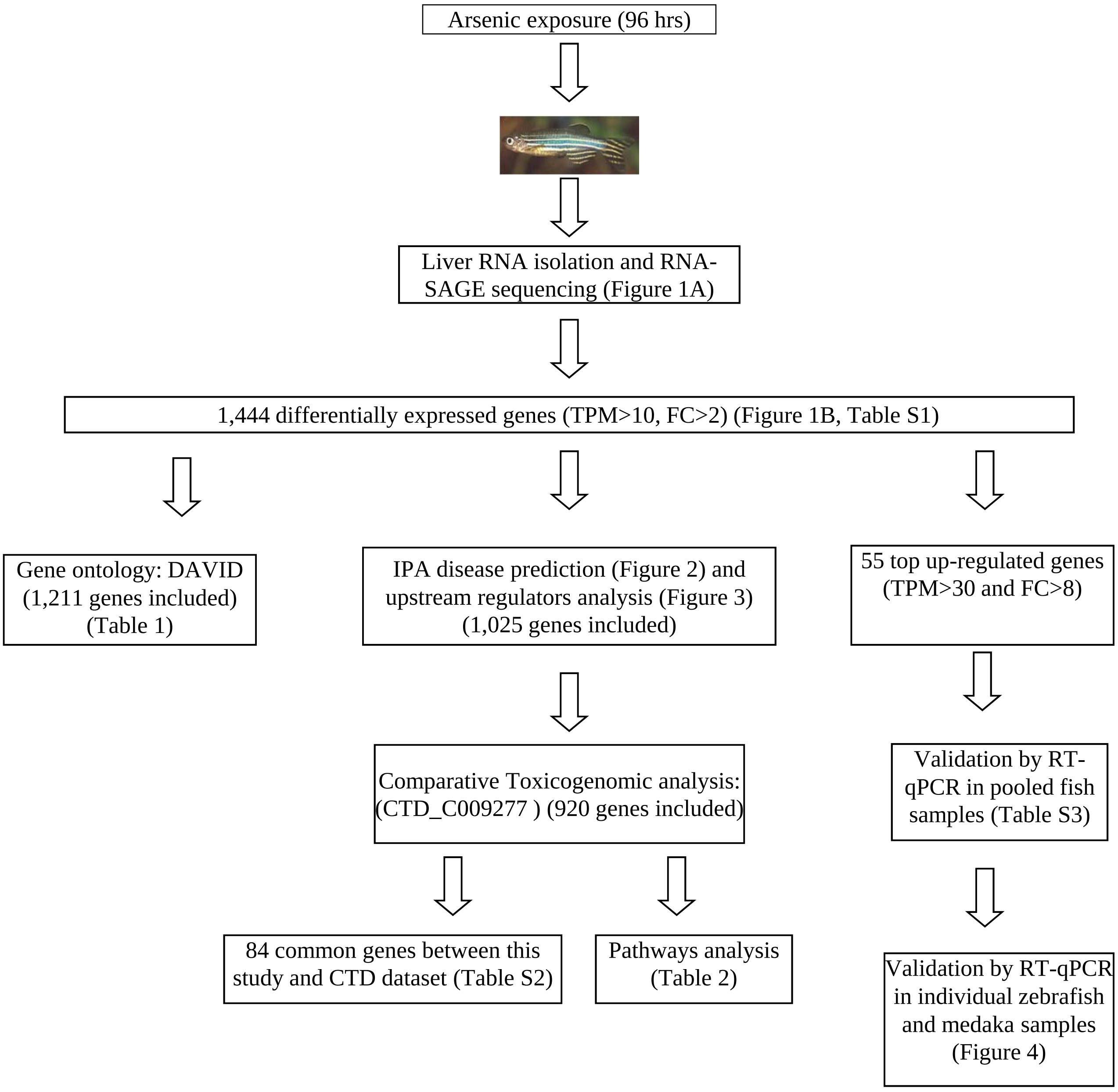

Figure S1. Flowchart of the study. Main steps and analyses are represented in each box in chronical order. Data generated from these analyses are shown in figures and tables as indicated in each box.
