# Supplementary material for: Genome-Wide Identification of Molecular Pathways and Biomarkers in Response to Arsenic Exposure in Zebrafish Liver
Source: PLoS One. 2013 Jul 29;8(7):e68737. doi: 10.1371/journal.pone.0068737 (PMC3726666; doi:10.1371/journal.pone.0068737)
Supplement: Table S3 — Up-regulated genes validated by qPCR using the same RNA sample for RNA sequencing. (DOCX) [file pone.0068737.s004.docx]

**Table S3. Up-regulated genes validated by qPCR using the same RNA sample for RNA sequencing**

| **GI** | **Refseq** | **Gene Symbol** | **RNA-SAGE** | | **qPCR** |
| --- | --- | --- | --- | --- | --- |
|  |  |  | **TPM^#^** | **log2 FC** | **-∆∆Ct** |
| 165972410* | NM_001113659 | *ferritin-like* | 2609.9 | 8.9 | 3.5 |
| 157954495 | NM_001109854 | *zgc:173594* | 1165.8 | 7.9 | 3.6 |
| 157841233* | NM_001109705 | *frh3* | 36.9 | 7.5 | 5.5 |
| 156616387* | NM_001102648 | *gstl* | 41.9 | 6.8 | 8.3 |
| 189514628* | XM_679483 | *adamtsl7* | 38.0 | 6.6 | 1.7 |
| 292620655* | XM_688586.4 | *es1l* | 801.0 | 5.4 | 6.9 |
| 41055404 | NM_200410 | *slc16a9a* | 193.6 | 5.2 | 7.9 |
| 157841229 | NM_001109722 | *cyp2aa7* | 186.8 | 5.1 | 1.8 |
| 117606288 | NM_001077604 | *klf11b* | 109.9 | 4.6 | 0.2 |
| 80751144 | NM_001037117 | *zgc:123218* | 44.8 | 4.5 | 4.4 |
| 42476266 | NM_131370 | *acat2* | 106.1 | 4.2 | 1.7 |
| 41393162 | NM_201503 | *mmp13a* | 65.6 | 4.1 | 0.8 |
| 292611118 | XM_002661794.1 |  | 8353.2 | 3.9 | 3.4 |
| 62955574 | NM_001017801 | *psmb4* | 116.7 | 3.8 | 0.9 |
| 288856245 | NM_001172308 |  | 46938.5 | 3.8 | 3.5 |
| 61806722 | NM_001013574 | *zgc:113516* | 78.0 | 3.7 | -0.2 |
| 307746870*^ | NM_001080034 | *mgstl* | 1407.4 | 3.6 | 4.8 |
| 157954451* | NM_001109832 | *cmbl* | 800.3 | 3.5 | 3.5 |
| 18858420 | NM_131187 | *ck2b* | 134.8 | 3.5 | 0.1 |
| 66472757 | NM_001020761 | *zgc:112089* | 114.9 | 3.5 | 0.3 |
| 47087300 | NM_213487 | *pitrm1* | 42.3 | 3.5 | 0.2 |
| 47085956 | NM_213175 | *epcam* | 67.6 | 3.5 | 0.8 |
| 18859378 | NM_131629 | *slc40a1* | 103.4 | 3.4 | 2.3 |
| 123706334 | NM_001080618 | *zgc:158580* | 5336.9 | 3.3 | 0.4 |
| 40363532 | NM_199212 | *sgk1* | 112.7 | 3.2 | 0.5 |
| 157311774* | NM_001105117 | *tm4sf4* | 114.7 | 3.2 | 2.5 |
| 114205449 | NM_001048055 | *lect2l* | 2157.9 | 3.1 | 3.1 |
| 50345087*^ | NM_001002215 | *mgst1* | 362.1 | 3.1 | 2.7 |
| 121583747 | NM_001079981 | *isoc1* | 335.9 | 3.1 | -0.4 |

*Manually annotated by sequence search in Genbank.

^#^TPM in the arsenic-treated sample.

^Both are homologs of *mgst1.*
